# Supplementary material for: Chemical and Pharmacological Profiling of Wrightia coccinea (Roxb. Ex Hornem.) Sims Focusing Antioxidant, Cytotoxic, Antidiarrheal, Hypoglycemic, and Analgesic Properties
Source: Molecules. 2022 Jun 22;27(13):4024. doi: 10.3390/molecules27134024 (PMC9268577; doi:10.3390/molecules27134024)
Supplement: Supplementary file 1 [file molecules-27-04024-s001.zip › molecules-1680918-supplementary.pdf]

Supplementary Files

Chemical and Pharmacological Profiling of *Wrightia coccinea* (roxb. Ex Hornem.) Sims Focusing Antioxidant, Cytotoxic, Antidiarrheal, Hypoglycemic, and Analgesic Properties

Tabassum Jannat [1,2], Md. Jamal Hossain [1,3,*], Ahmed M. El-Shehawi [4], Md. Ruhul Kuddus [1], Mohammad A. Rashid [1], Sarah Albogami [4], Ibrahim Jafri [4], Mohamed El-Shazly [5,6] and Mohammad Rashedul Haque [1,*]

[1] Phytochemical Research Laboratory, Department of Pharmaceutical Chemistry, Faculty of Pharmacy, University of Dhaka, Dhaka 1000, Bangladesh
[2] Department Pharmacy, University of Asia Pacific, Dhaka 1205, Bangladesh
[3] Department of Pharmacy, State University of Bangladesh, 77 Satmasjid Road, Dhanmondi, Dhaka 1205, Bangladesh
[4] Department of Biotechnology, College of Science, Taif University, P.O. Box 11099, Taif 21944, Saudi Arabia
[5] Department of Pharmacognosy, Faculty of Pharmacy, Ain Shams University, Cairo 11566, Egypt
[6] Department of Pharmaceutical Biology, Faculty of Pharmacy and Biotechnology, German University in Cairo (GUC), Cairo 11835, Egypt

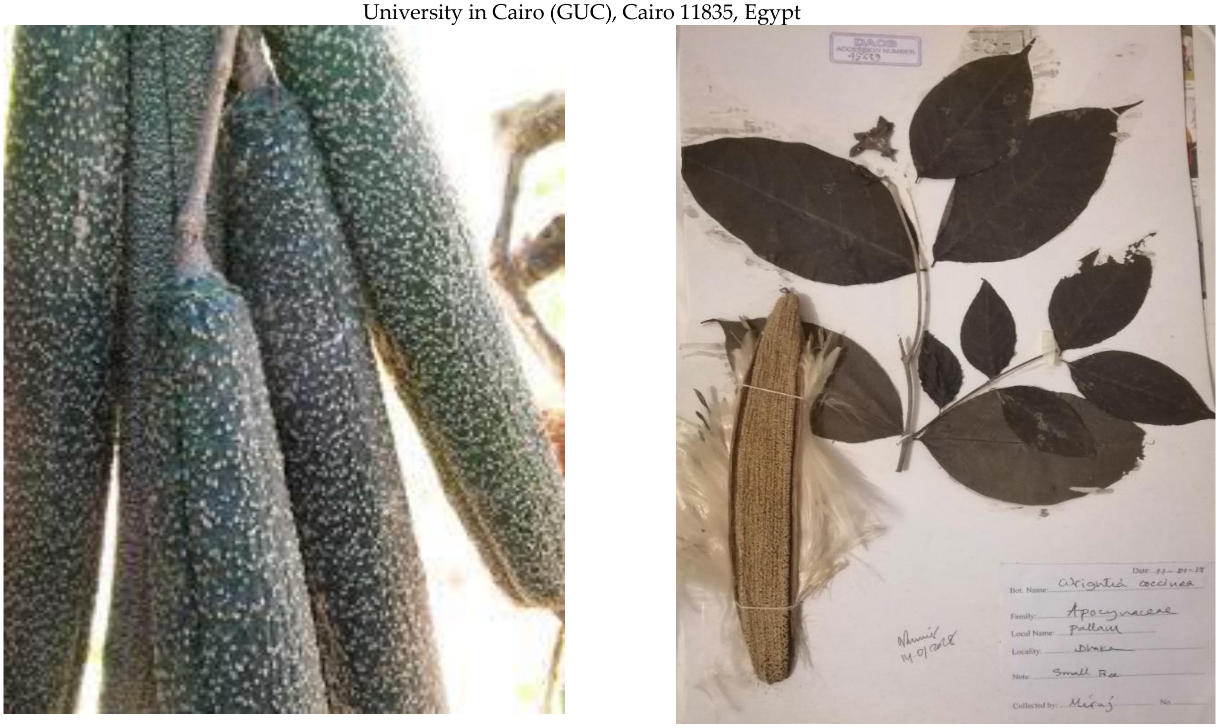

(a) **(b)**

Figure S1. Pictures of *Wrightia coccinea*: (**a**) Fruit part, (**b**) Voucher specimen for identification of collected *Wrightia coccinea* by Bangladesh National Herbarium with its accession number 45689.

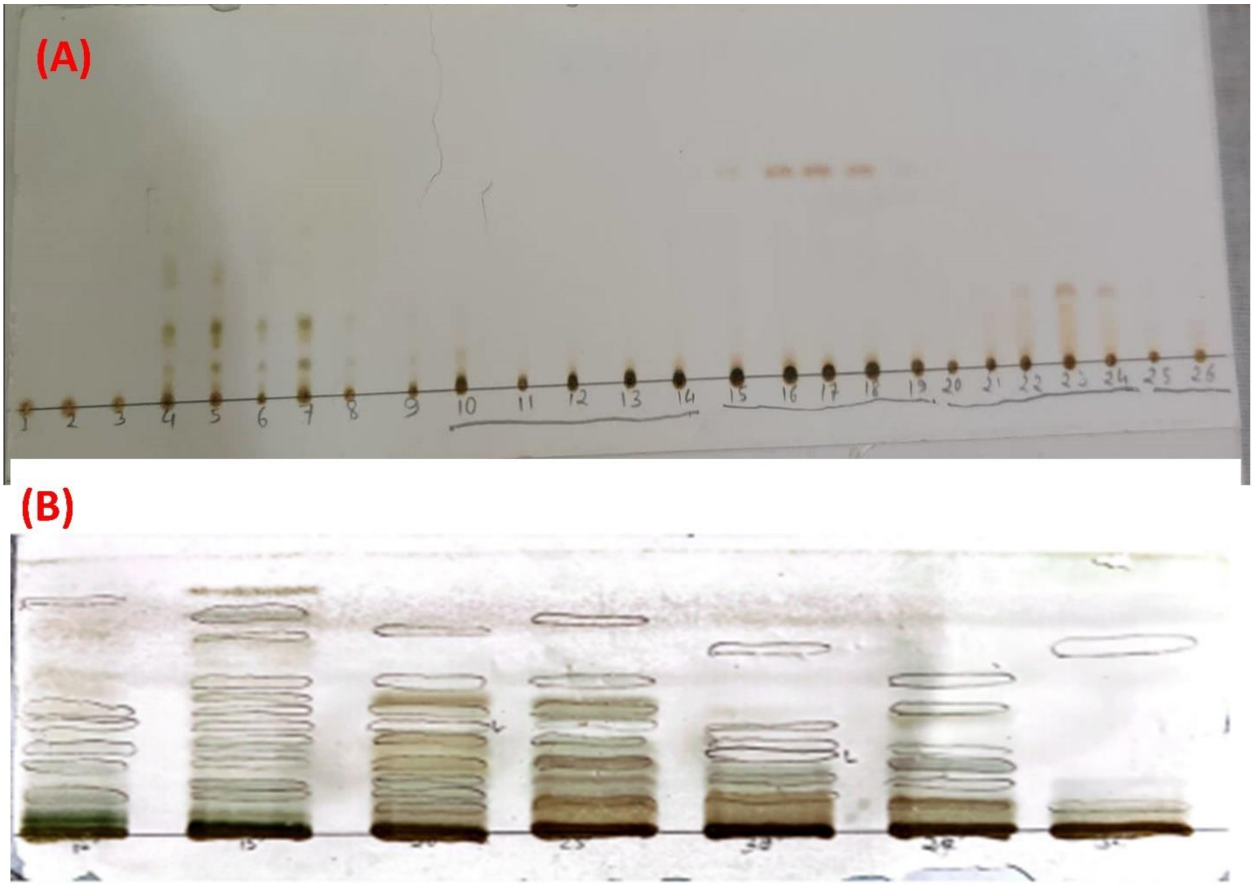

Figure S2: (A) Initial Screening of the VLC fractions of *Wrightia coccinea* (10% ethyl acetate in toluene), **(B)** screening of the mixed similar beakers VLC fractions of *Wrightia coccinea* (15% ethyl acetate in toluene).

Table S1. Different solvent fraction used for VLC analysis of crude methanol extract of bark of *W. coccinea.*

Beaker Number	Solvent System	Volume Collected
1	100 % Hexane	150 mL
3	2.5 % Ethyl Acetate in Hexane	150 mL
4	5 % Ethyl Acetate in Hexane	150 mL
5	7.5 % Ethyl Acetate in Hexane	150 mL
6	10 % Ethyl Acetate in Hexane	150 mL
7	12.5 % Ethyl Acetate in Hexane	150 mL
8	15 % Ethyl Acetate in Hexane	150 mL
9	17.5% Ethyl Acetate in Hexane	150 mL
10	20 % Ethyl Acetate in Hexane	150 mL

11	22.5% Ethyl Acetate in Hexane	150 mL
12	25 % Ethyl Acetate in Hexane	150 mL
13	27.5% Ethyl Acetate in Hexane	150 mL
14	30 % Ethyl Acetate in Hexane	150 mL
15	35 % Ethyl Acetate in Hexane	150 mL
16	40 % Ethyl Acetate in Hexane	150 mL
17	45 % Ethyl Acetate in Hexane	150 mL
18	50 % Ethyl Acetate in Hexane	150 mL
19	60 % Ethyl Acetate in Hexane	150 mL
20	70 % Ethyl Acetate in Hexane	150 mL
21	80 % Ethyl Acetate in Hexane	150 mL
22	100 % Ethyl Acetate	100 mL
23	5 % Methanol in Ethyl Acetate	100 mL
24	10 % Methanol in Ethyl Acetate	100 mL
25	20 % Methanol in Ethyl Acetate	100 mL
26	30 % Methanol in Ethyl Acetate	100 mL

Table S2. Preparative Thin Layer Chromatography (PTLC) of selected fractions from Size Exclusion Chromatography (SEC) of different VLC fractions of *W. coccinea*.

**Fraction**	**Solvent System**	**UV**		**Spray Color**	**Sample ID**
		**Short**	**Long**		
4-8 (beaker direct from VLC)	Ethyl acetate: Petroleum ether= 5:95)	Yes	No	Brown	Vial-A
		Yes	No	Violet	WC-1
		No	Yes	Ash	WC-2
9-16	Ethyl acetate: Hexane= 5:95)	No	No	Violet	WC-3
		No	Yes	Yellow	WC-4
		Yes	No	Yellow	WC-5
		Yes	No	Pink	WC-6
17-22	Chloroform: Hexane= 80:20)	No	No	Violet	WC-11
23-28	Chloroform: Hexane= 80:20)	No	No	Purple	WC-7

		No	Yes	Violet	WC-8
		Yes	Yes	-	WC-9
		Yes	Yes	-	Vial-37
29-33	Chloroform: Hexane= 80:20)	Yes	No	Dark violet	Vial-38
		No	Yes	-	WC-10

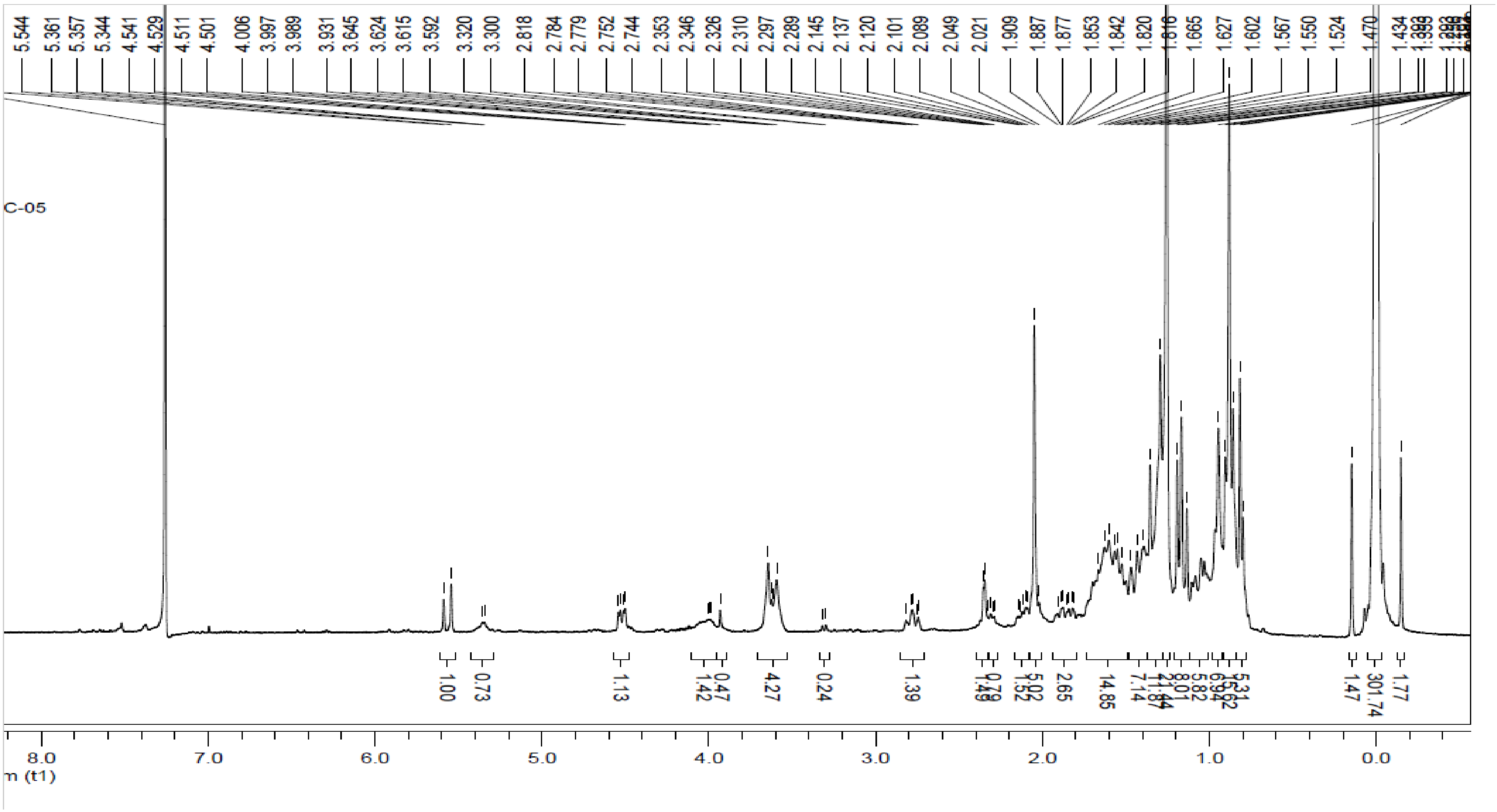

Figure S3: ^{1}H-NMR spectrum of compound 1 (3β-acetyloxy-olean-12-en-28-ol).

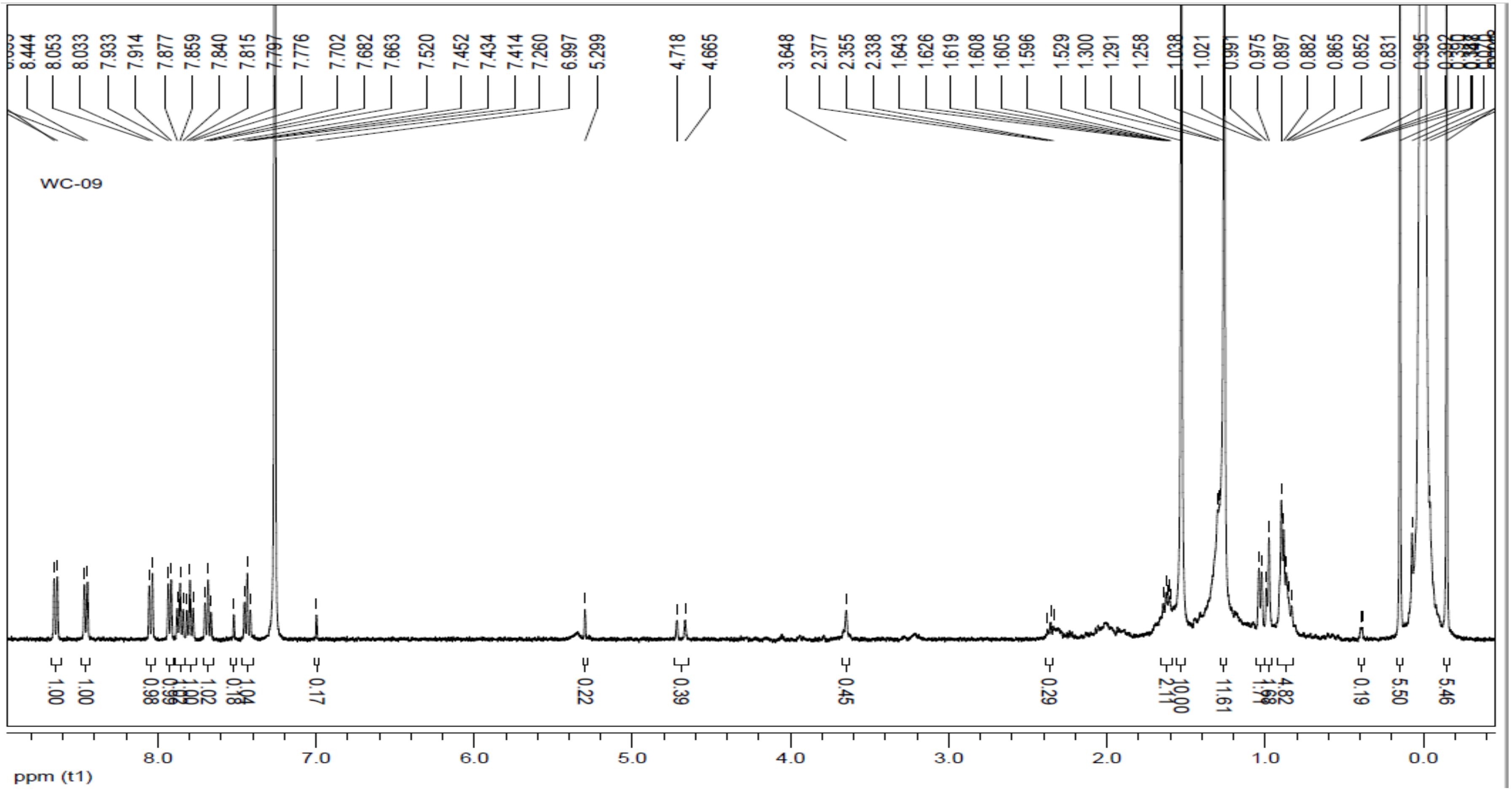

Figure S4: ^{1}H-NMR spectrum of compound 2 (Wrightiadione).

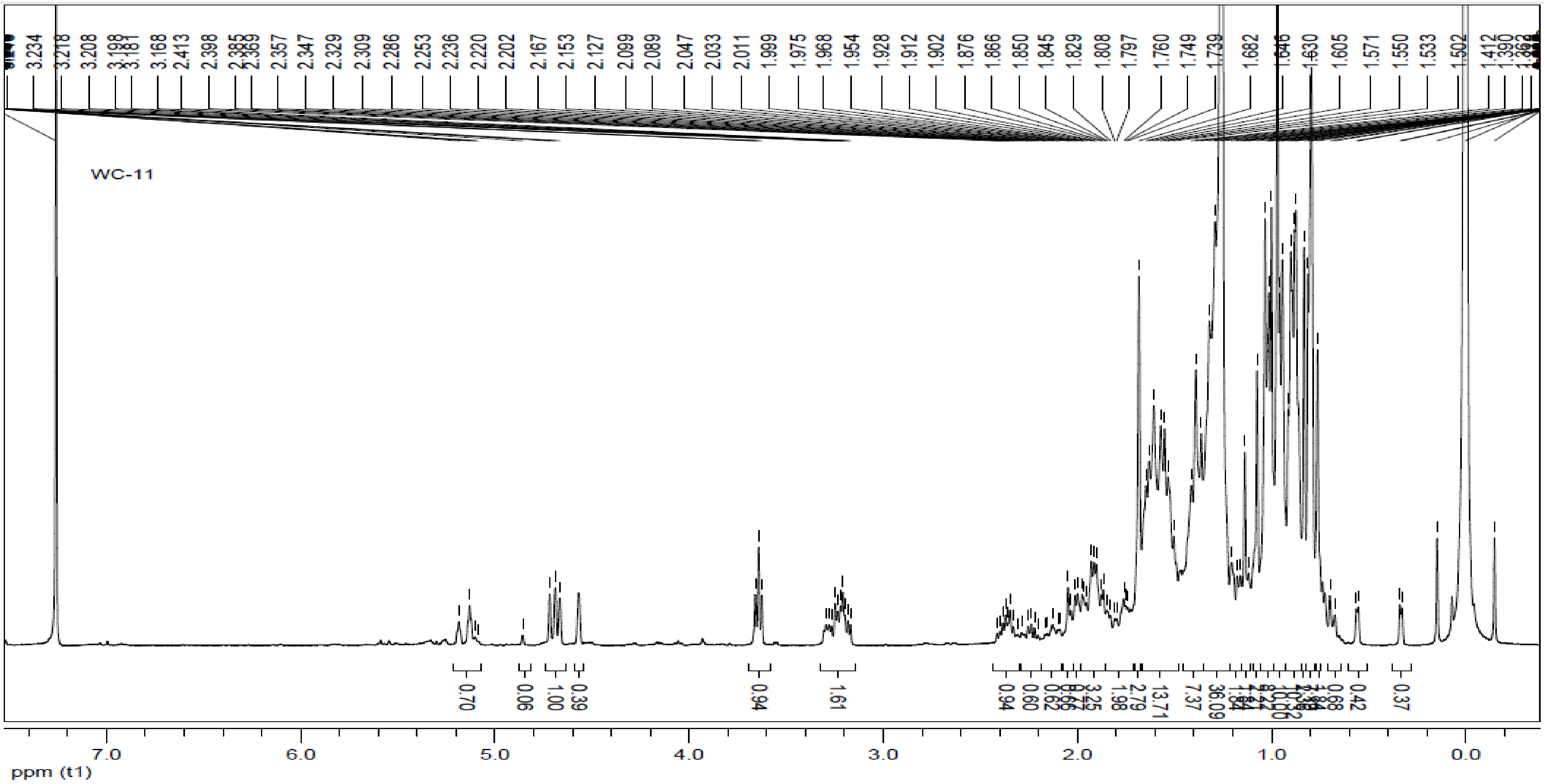

Figure S5: ^{1}H-NMR spectrum of compound 3 (22β-Hydroxylupeol).

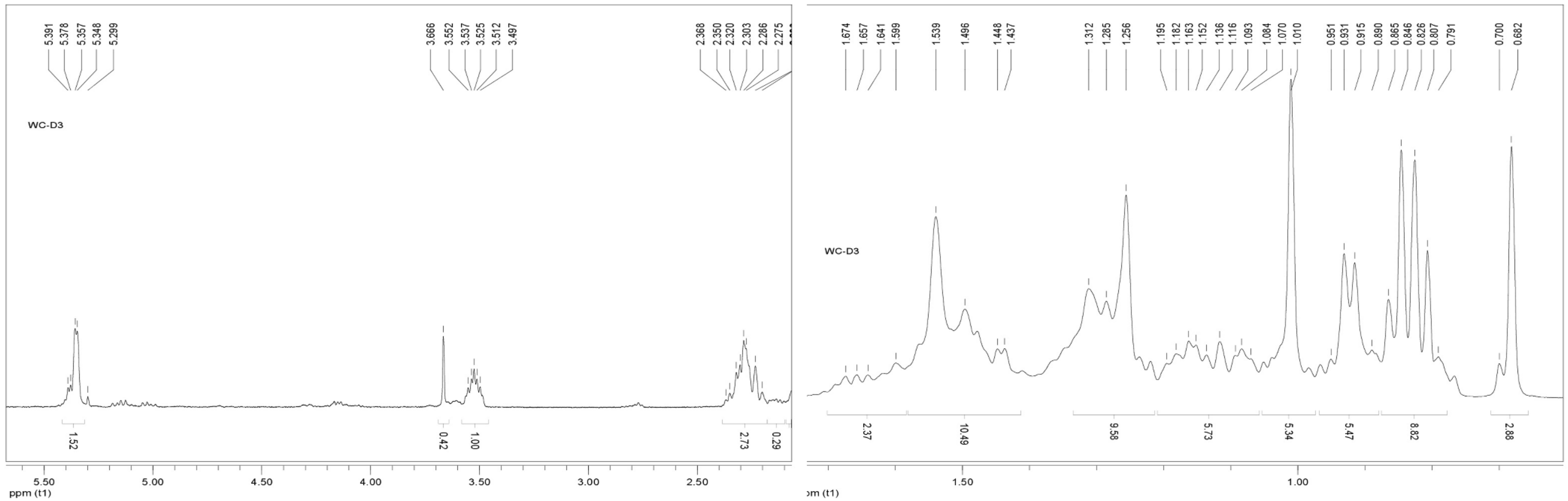

Figure S6: ^{1}H-NMR spectrum of compound 4 (β-Sitosterol).

Table S3. Molecular docking analysis of the isolated compounds (1 to 4) from *Wrightia coccinea* and the standard compound Butylated Hydroxy toluene (BHT) against the glutathione reductase enzyme (PDB ID: 3GRS).

Compounds	Bond (AA and Ligand)	Bond length (Å)	Bond type	Bond nature
3β-acetyloxy-olean-12-en-28-ol (Compound 1)	A:GLY290:HN - N:UNK1:O	2.63366	Hydrogen Bond	Conventional Hydrogen Bond
	N:UNK1:C - A:PHE226	3.55429	Hydrophobic	Pi-Sigma
	A:LEU337 - N:UNK1	5.01904	Hydrophobic	Alkyl
	N:UNK1:C - A:ARG291	3.64022	Hydrophobic	Alkyl
	N:UNK1:C - A:LEU337	4.82873	Hydrophobic	Alkyl
	N:UNK1:C - A:ILE198	4.35628	Hydrophobic	Alkyl
	N:UNK1:C - A:ARG291	4.6948	Hydrophobic	Alkyl
	A:TYR197 - N:UNK1	5.27606	Hydrophobic	Pi-Alkyl
	A:TYR197 - N:UNK1:C	5.44626	Hydrophobic	Pi-Alkyl
	A:TYR197 - N:UNK1:C	4.39683	Hydrophobic	Pi-Alkyl
	A:PHE226 - N:UNK1:C	5.18396	Hydrophobic	Pi-Alkyl
	A:PHE226 - N:UNK1	5.24516	Hydrophobic	Pi-Alkyl
Wrightiadione (Compound 2)	A:LYS66:HZ1 - N:UNK1:O	2.30693	Hydrogen Bond	Conventional Hydrogen Bond
	A:LYS66:HZ3 - N:UNK1:O	2.44677	Hydrogen Bond	Conventional Hydrogen Bond
	A:LYS66:HZ3 - N:UNK1:O	2.19345	Hydrogen Bond	Conventional Hydrogen Bond
	A:ILE198:CD - N:UNK1	3.79877	Hydrophobic	Pi-Sigma
	A:CYS63:SG - N:UNK1	3.53928	Other	Pi-Sulfur
	A:TYR197 - N:UNK1	5.31147	Hydrophobic	Pi-Pi T-shaped
	N:UNK1 - A:TYR197	5.4611	Hydrophobic	Pi-Pi T-shaped
	A:GLY62:C,O;CYS63:N - N:UNK1	4.1532	Hydrophobic	Amide-Pi Stacked
	A:GLY62:C,O;CYS63:N - N:UNK1	4.57027	Hydrophobic	Amide-Pi Stacked
	N:UNK1 - A:CYS63	4.11999	Hydrophobic	Pi-Alkyl
	N:UNK1 - A:ILE198	5.15683	Hydrophobic	Pi-Alkyl
	N:UNK1 - A:LEU338	5.23607	Hydrophobic	Pi-Alkyl
22β-hydroxylupeol (Compound 3)	A:ALA336 - N:UNK1	4.74846	Hydrophobic	Alkyl
	A:ALA336 - N:UNK1:C	4.36408	Hydrophobic	Alkyl
	A:LEU337 - N:UNK1	4.88381	Hydrophobic	Alkyl
	A:LEU338 - N:UNK1	5.18816	Hydrophobic	Alkyl
	N:UNK1:C - A:ARG291	4.19248	Hydrophobic	Alkyl
	N:UNK1:C - A:LEU337	4.81402	Hydrophobic	Alkyl
	N:UNK1:C - A:ILE198	4.73086	Hydrophobic	Alkyl

	N:UNK1:C - A:ARG291	4.66269	Hydrophobic	Alkyl
	A:TYR197 - N:UNK1	5.37881	Hydrophobic	Pi-Alkyl
	A:TYR197 - N:UNK1	5.11012	Hydrophobic	Pi-Alkyl
	A:TYR197 - N:UNK1:C	4.55992	Hydrophobic	Pi-Alkyl
	A:PHE226 - N:UNK1:C	4.20172	Hydrophobic	Pi-Alkyl
	A:PHE226 - N:UNK1:C	3.63857	Hydrophobic	Pi-Alkyl
β-sitosterol (Compound 4)	N:UNK1:H - A:GLY381:O	2.18384	Hydrogen Bond	Conventional Hydrogen Bond
	A:LEU338 - N:UNK1	5.32242	Hydrophobic	Alkyl
	N:UNK1 - A:LEU337	5.4098	Hydrophobic	Alkyl
	N:UNK1:C - A:ILE198	4.49934	Hydrophobic	Alkyl
	N:UNK1:C - A:ARG291	4.58556	Hydrophobic	Alkyl
	A:TYR197 - N:UNK1	5.48427	Hydrophobic	Pi-Alkyl
	A:TYR197 - N:UNK1	4.80576	Hydrophobic	Pi-Alkyl
	A:PHE226 - N:UNK1:C	4.20625	Hydrophobic	Pi-Alkyl
Butylated Hydroxy toluene (BHT)	N:UNK1:H - A:GLY158:O	2.64302	Hydrogen Bond	Conventional Hydrogen Bond
	N:UNK1:C - A:LYS53	4.40683	Hydrophobic	Alkyl
	N:UNK1:C - A:VAL61	4.83698	Hydrophobic	Alkyl

Table S4. Molecular docking analysis of the isolated compounds (1 to 4) from *Wrightia coccinea* and the standard compound vincristine against the epidermal growth factor receptor (EGFR) (PDB ID: 1XKK).

Compounds	Bond (AA and Ligand)	Bond length (Å)	Bond type	Bond nature
3β-acetyloxy-olean-12-en-28-ol (Compound 1)	A:ARG803:HH21 - N:UNK1:O	2.50757	Hydrogen Bond	Conventional Hydrogen Bond
	N:UNK1:H - A:ASP855:OD2	2.85203	Hydrogen Bond	Conventional Hydrogen Bond
	A:VAL726 - N:UNK1	4.97025	Hydrophobic	Alkyl
	A:VAL726 - N:UNK1	4.61711	Hydrophobic	Alkyl
	A:VAL726 - N:UNK1	4.83932	Hydrophobic	Alkyl
	A:ALA743 - N:UNK1	4.22256	Hydrophobic	Alkyl
	A:ALA743 - N:UNK1:C	4.30025	Hydrophobic	Alkyl
	A:ALA743 - N:UNK1:C	2.45095	Hydrophobic	Alkyl
	A:CYS797 - N:UNK1	5.00788	Hydrophobic	Alkyl
	A:ARG841 - N:UNK1	4.76636	Hydrophobic	Alkyl
	A:LEU844 - N:UNK1	4.97267	Hydrophobic	Alkyl
	A:LEU844 - N:UNK1	4.21999	Hydrophobic	Alkyl
	N:UNK1:C - A:CYS797	3.3151	Hydrophobic	Alkyl
	N:UNK1:C - A:LEU844	5.19677	Hydrophobic	Alkyl
	N:UNK1:C - A:LEU799	4.90459	Hydrophobic	Alkyl

	N:UNK1:C - A:ARG841	4.74919	Hydrophobic	Alkyl
	N:UNK1:C - A:LEU792	5.21087	Hydrophobic	Alkyl
	N:UNK1:C - A:MET793	5.05951	Hydrophobic	Alkyl
	N:UNK1:C - A:LEU844	3.96732	Hydrophobic	Alkyl
	N:UNK1:C - A:LEU718	4.66308	Hydrophobic	Alkyl
	N:UNK1:C - A:VAL726	4.50838	Hydrophobic	Alkyl
	N:UNK1:C - A:LEU792	5.41707	Hydrophobic	Alkyl
Wrightiadione (Compound 2)	A:MET793:HN - N:UNK1:O	2.08632	Hydrogen Bond	Conventional Hydrogen Bond
	A:LEU718:CD1 - N:UNK1	3.63663	Hydrophobic	Pi-Sigma
	A:VAL726:CG1 - N:UNK1	3.94659	Hydrophobic	Pi-Sigma
	A:LEU844:CD1 - N:UNK1	3.91358	Hydrophobic	Pi-Sigma
	A:LEU844:CD2 - N:UNK1	3.9219	Hydrophobic	Pi-Sigma
	N:UNK1 - A:LEU718	5.03715	Hydrophobic	Pi-Alkyl
	N:UNK1 - A:VAL726	5.04011	Hydrophobic	Pi-Alkyl
	N:UNK1 - A:ALA743	4.22622	Hydrophobic	Pi-Alkyl
	N:UNK1 - A:ALA743	4.27107	Hydrophobic	Pi-Alkyl
	N:UNK1 - A:LEU844	5.42689	Hydrophobic	Pi-Alkyl
	N:UNK1 - A:VAL726	4.85229	Hydrophobic	Pi-Alkyl
	N:UNK1 - A:ALA743	5.38401	Hydrophobic	Pi-Alkyl
22β-hydroxylupeol (Compound 3)	A:THR854:HG1 - N:UNK1:O	2.03973	Hydrogen Bond	Conventional Hydrogen Bond
	A:VAL726 - N:UNK1	4.60881	Hydrophobic	Alkyl
	A:ALA743 - N:UNK1:C	3.98785	Hydrophobic	Alkyl
	A:CYS797 - N:UNK1	5.02341	Hydrophobic	Alkyl
	A:ARG841 - N:UNK1	5.04967	Hydrophobic	Alkyl
	A:LEU844 - N:UNK1	5.10522	Hydrophobic	Alkyl
	N:UNK1:C - A:CYS797	4.43513	Hydrophobic	Alkyl
	N:UNK1:C - A:LEU844	4.63146	Hydrophobic	Alkyl
	N:UNK1:C - A:VAL726	3.49641	Hydrophobic	Alkyl
	N:UNK1:C - A:LYS745	4.31664	Hydrophobic	Alkyl
	N:UNK1:C - A:ARG841	3.76828	Hydrophobic	Alkyl
	N:UNK1:C - A:LEU792	5.04719	Hydrophobic	Alkyl
	N:UNK1:C - A:MET793	4.9517	Hydrophobic	Alkyl
	N:UNK1:C - A:LEU844	4.10445	Hydrophobic	Alkyl
	N:UNK1:C - A:LEU718	3.76732	Hydrophobic	Alkyl
	N:UNK1:C - A:VAL726	5.15238	Hydrophobic	Alkyl
β-sitosterol (Compound 4)	A:LEU718 - N:UNK1	4.98581	Hydrophobic	Alkyl
	A:LEU718 - N:UNK1	3.79747	Hydrophobic	Alkyl
	A:VAL726 - N:UNK1	4.64438	Hydrophobic	Alkyl
	A:VAL726 - N:UNK1	3.92006	Hydrophobic	Alkyl
	A:ALA743 - N:UNK1	3.91507	Hydrophobic	Alkyl
	A:LEU844 - N:UNK1	4.59947	Hydrophobic	Alkyl

	N:UNK1:C - A:LEU844	3.76244	Hydrophobic	Alkyl
	N:UNK1 - A:LEU718	4.34623	Hydrophobic	Alkyl
	N:UNK1:C - A:CYS797	3.47894	Hydrophobic	Alkyl
	N:UNK1:C - A:VAL726	4.58958	Hydrophobic	Alkyl
	N:UNK1:C - A:LYS745	4.12078	Hydrophobic	Alkyl
	N:UNK1:C - A:MET766	4.84806	Hydrophobic	Alkyl
	N:UNK1:C - A:LEU777	4.18583	Hydrophobic	Alkyl
Vincristine	A:CYS797:HN - N:UNK1:O	2.913	Hydrogen Bond	Conventional Hydrogen Bond
	A:GLY721:CA - N:UNK1:O	3.62858	Hydrogen Bond	Carbon Hydrogen Bond
	A:ARG841:CD - N:UNK1:O	3.492	Hydrogen Bond	Carbon Hydrogen Bond
	A:ALA722 - N:UNK1:C	3.72581	Hydrophobic	Alkyl
	A:CYS797 - N:UNK1	5.46659	Hydrophobic	Alkyl
	N:UNK1:C - A:PRO877	4.38162	Hydrophobic	Alkyl
	N:UNK1:C - A:LEU799	5.22758	Hydrophobic	Alkyl
	N:UNK1:C - A:CYS797	3.34519	Hydrophobic	Alkyl
	N:UNK1:C - A:VAL726	4.12815	Hydrophobic	Alkyl

Table S5. Molecular docking analysis of the isolated compounds (1 to 4) from *Wrightia coccinea* and the standard compound loperamide against the kappa opioid receptor (PDB ID: 6VI4).

Compounds	Bond (AA and Ligand)	Bond length (Å)	Bond type	Bond nature
3β-acetyloxy-olean-12-en-28-ol (Compound 1)	B:SER136:HG - N:UNK1:O	2.39948	Hydrogen Bond	Conventional Hydrogen Bond
	B:TRP183:CD1 - N:UNK1:O	3.38888	Hydrogen Bond	Carbon Hydrogen Bond
	N:UNK1:C - B:LEU103	4.97783	Hydrophobic	Alkyl
	B:PHE99 - N:UNK1:C	5.1512	Hydrophobic	Pi-Alkyl
	B:PHE99 - N:UNK1:C	4.92899	Hydrophobic	Pi-Alkyl
	B:TYR140 - N:UNK1:C	4.81273	Hydrophobic	Pi-Alkyl
	B:TRP183 - N:UNK1	4.46384	Hydrophobic	Pi-Alkyl
	B:TRP183 - N:UNK1	5.46122	Hydrophobic	Pi-Alkyl
	B:TRP183 - N:UNK1	4.51067	Hydrophobic	Pi-Alkyl
Wrightiadione (Compound 2)	B:TRP183 - N:UNK1	3.84494	Hydrophobic	Pi-Pi Stacked
	B:TRP183 - N:UNK1	4.26419	Hydrophobic	Pi-Pi Stacked
	B:TRP183 - N:UNK1	4.65623	Hydrophobic	Pi-Pi Stacked
	B:TRP183 - N:UNK1	4.4352	Hydrophobic	Pi-Pi Stacked
	B:TRP183 - N:UNK1	4.5261	Hydrophobic	Pi-Pi Stacked
	N:UNK1 - B:TRP183	3.72894	Hydrophobic	Pi-Pi Stacked
	N:UNK1 - B:ILE180	5.14429	Hydrophobic	Pi-Alkyl
22β-hydroxylupeol (Compound 3)	N:UNK1:C - B:TRP183	3.67657	Hydrophobic	Pi-Sigma
	N:UNK1:C - B:ILE133	4.1537	Hydrophobic	Alkyl
	N:UNK1:C - B:ILE133	3.9069	Hydrophobic	Alkyl
	N:UNK1:C - B:ILE137	4.60114	Hydrophobic	Alkyl

	N:UNK1:C - B:LEU184	5.48848	Hydrophobic	Alkyl
	B:TRP183 - N:UNK1	4.63115	Hydrophobic	Pi-Alkyl
	B:TRP183 - N:UNK1	4.80502	Hydrophobic	Pi-Alkyl
	B:TRP183 - N:UNK1:C	5.18799	Hydrophobic	Pi-Alkyl
	B:TRP183 - N:UNK1	5.35166	Hydrophobic	Pi-Alkyl
	B:TRP183 - N:UNK1:C	5.39785	Hydrophobic	Pi-Alkyl
β-sitosterol (Compound 4)	B:SER136:HG - N:UNK1:O	2.76756	Hydrogen Bond	Conventional Hydrogen Bond
	N:UNK1:C - B:ILE180	3.99865	Hydrophobic	Alkyl
	N:UNK1:C - B:ILE180	5.06823	Hydrophobic	Alkyl
	B:TYR140 - N:UNK1	5.2348	Hydrophobic	Pi-Alkyl
	B:TYR140 - N:UNK1	4.80032	Hydrophobic	Pi-Alkyl
	B:TRP183 - N:UNK1	3.81076	Hydrophobic	Pi-Alkyl
	B:TRP183 - N:UNK1	5.41333	Hydrophobic	Pi-Alkyl
	B:TRP183 - N:UNK1:C	5.14163	Hydrophobic	Pi-Alkyl
	B:TRP183 - N:UNK1	4.37087	Hydrophobic	Pi-Alkyl
	B:TRP183 - N:UNK1	5.13248	Hydrophobic	Pi-Alkyl
	B:TRP183 - N:UNK1	5.29101	Hydrophobic	Pi-Alkyl
Loperamide	N:UNK1:C - B:TRP183	3.49962	Hydrophobic	Pi-Sigma
	B:TRP183 - N:UNK1	4.98817	Hydrophobic	Pi-Pi Stacked
	N:UNK1:Cl - B:ILE96	4.98775	Hydrophobic	Alkyl
	B:PHE99 - N:UNK1:Cl	5.44338	Hydrophobic	Pi-Alkyl
	B:TRP183 - N:UNK1	4.90493	Hydrophobic	Pi-Alkyl
	N:UNK1 - B:LEU107	5.32138	Hydrophobic	Pi-Alkyl

Table S6. Molecular docking analysis of the isolated compounds (1 to 4) from *Wrightia coccinea* and the standard compound glibenclamide against the glucose transporter 3 (GLUT 3) (PDB ID: 4ZWB).

**Compounds**	**Bond (AA and Ligand)**	**Bond length (Å)**	**Bond type**	**Bond nature**
3β-acetyloxy-olean-12-en-28-ol (Compound 1)	N:UNK1:H - A:LEU193:O	2.37922	Hydrogen Bond	Conventional Hydrogen Bond
	A:ILE19 - N:UNK1	4.57521	Hydrophobic	Alkyl
	A:PRO194 - N:UNK1	4.48281	Hydrophobic	Alkyl
	N:UNK1:C - A:ILE19	4.92455	Hydrophobic	Alkyl
	A:PHE22 - N:UNK1	4.55047	Hydrophobic	Pi-Alkyl
	A:PHE22 - N:UNK1:C	4.39087	Hydrophobic	Pi-Alkyl
	A:PHE22 - N:UNK1:C	4.93416	Hydrophobic	Pi-Alkyl
	A:PHE190 - N:UNK1	5.40289	Hydrophobic	Pi-Alkyl
Wrightiadione (Compound 2)	A:GLN281:HE21 - N:UNK1:O	2.25454	Hydrogen Bond	Conventional Hydrogen Bond
	A:ASN413:HD21 - N:UNK1:O	2.35664	Hydrogen Bond	Conventional Hydrogen Bond
	A:THR28:CG2 - N:UNK1	3.67058	Hydrophobic	Pi-Sigma
	N:UNK1 - A:ILE162	5.34165	Hydrophobic	Pi-Alkyl
22β-hydroxylupeol (Compound 3)	N:UNK1:C - A:PHE22	3.44725	Hydrophobic	Pi-Sigma
	A:ILE19 - N:UNK1	5.09638	Hydrophobic	Alkyl

	A:PRO194 - N:UNK1	4.80434	Hydrophobic	Alkyl
	A:LEU197 - N:UNK1	4.93286	Hydrophobic	Alkyl
	N:UNK1 - A:LEU197	5.07074	Hydrophobic	Alkyl
	N:UNK1:C - A:VAL164	4.12175	Hydrophobic	Alkyl
	N:UNK1:C - A:LEU193	4.6634	Hydrophobic	Alkyl
	N:UNK1:C - A:PRO194	4.55645	Hydrophobic	Alkyl
	N:UNK1:C - A:LEU193	5.29054	Hydrophobic	Alkyl
	N:UNK1:C - A:LEU197	3.88069	Hydrophobic	Alkyl
	A:PHE22 - N:UNK1:C	5.20055	Hydrophobic	Pi-Alkyl
	A:PHE190 - N:UNK1:C	4.76624	Hydrophobic	Pi-Alkyl
β-sitosterol (Compound 4)	N:UNK1:C - A:TYR290	3.82548	Hydrophobic	Pi-Sigma
	N:UNK1:C - A:TYR290	3.88045	Hydrophobic	Pi-Sigma
	A:ILE166 - N:UNK1	4.31775	Hydrophobic	Alkyl
	N:UNK1 - A:ILE166	5.48538	Hydrophobic	Alkyl
	N:UNK1 - A:ILE285	4.53105	Hydrophobic	Alkyl
	N:UNK1:C - A:ILE285	3.39383	Hydrophobic	Alkyl
	N:UNK1:C - A:VAL67	4.80888	Hydrophobic	Alkyl
	N:UNK1:C - A:VAL67	5.44744	Hydrophobic	Alkyl
	A:PHE289 - N:UNK1	4.14384	Hydrophobic	Pi-Alkyl
Glibenclamide	A:ASN32:HD21 - N:UNK1:O	2.20588	Hydrogen Bond	Conventional Hydrogen Bond
	A:ASN286:HD21 - N:UNK1:O	2.42121	Hydrogen Bond	Conventional Hydrogen Bond
	N:UNK1:H - A:VAL67:O	2.42244	Hydrogen Bond	Conventional Hydrogen Bond
	A:ASN286:CA - N:UNK1:O	3.75651	Hydrogen Bond	Carbon Hydrogen Bond
	N:UNK1:C - A:ASN286:OD1	3.66366	Hydrogen Bond	Carbon Hydrogen Bond
	A:GLY417:C,O;LEU418:N - N:UNK1	4.08507	Hydrophobic	Amide-Pi Stacked
	A:ALA68 - N:UNK1:Cl	3.67527	Hydrophobic	Alkyl
	A:ILE285 - N:UNK1	5.23041	Hydrophobic	Alkyl
	N:UNK1:Cl - A:LEU418	4.09905	Hydrophobic	Alkyl
	A:TYR290 - N:UNK1:C	4.04738	Hydrophobic	Pi-Alkyl
	A:PHE414 - N:UNK1:Cl	4.17738	Hydrophobic	Pi-Alkyl
	N:UNK1 - A:VAL67	5.2835	Hydrophobic	Pi-Alkyl
	N:UNK1 - A:ALA68	4.56263	Hydrophobic	Pi-Alkyl

Table S7. Molecular docking analysis of the isolated compounds (1 to 4) from *Wrightia coccinea* and the standard compound morphine against the Mu-opioid receptor (PDB ID: 5C1M).

Compounds	Bond (AA and Ligand)	Bond length (Å)	Bond type	Bond nature
3β-acetyloxy-olean-12-en-28-ol (Compound 1)	N:UNK1:C - A:HIS54	3.59197	Hydrophobic	Pi-Sigma
	A:ILE296 - N:UNK1	4.78447	Hydrophobic	Alkyl
	A:VAL300 - N:UNK1	4.1404	Hydrophobic	Alkyl
	A:ILE322 - N:UNK1	4.86088	Hydrophobic	Alkyl

	A:ILE322 - N:UNK1	5.34218	Hydrophobic	Alkyl
	N:UNK1:C - A:MET151	5.18904	Hydrophobic	Alkyl
	N:UNK1:C - A:VAL236	3.24948	Hydrophobic	Alkyl
	N:UNK1:C - A:ILE322	5.14503	Hydrophobic	Alkyl
	N:UNK1:C - A:ILE322	4.51331	Hydrophobic	Alkyl
	A:HIS54 - N:UNK1	5.13372	Hydrophobic	Pi-Alkyl
	A:HIS54 - N:UNK1:C	4.43051	Hydrophobic	Pi-Alkyl
	A:TYR75 - N:UNK1:C	4.76864	Hydrophobic	Pi-Alkyl
	A:TYR148 - N:UNK1	5.29743	Hydrophobic	Pi-Alkyl
	A:TYR148 - N:UNK1:C	4.97909	Hydrophobic	Pi-Alkyl
	A:HIS297 - N:UNK1	5.01978	Hydrophobic	Pi-Alkyl
	A:TRP318 - N:UNK1:C	4.74024	Hydrophobic	Pi-Alkyl
	A:TRP318 - N:UNK1:C	5.16765	Hydrophobic	Pi-Alkyl
	A:HIS319 - N:UNK1:C	4.10098	Hydrophobic	Pi-Alkyl
	A:HIS319 - N:UNK1:C	4.90845	Hydrophobic	Pi-Alkyl
Wrightiadione (Compound 2)	A:HIS54:CE1 - N:UNK1:O	3.52447	Hydrogen Bond	Carbon Hydrogen Bond
	A:ASP147:OD2 - N:UNK1	4.27979	Electrostatic	Pi-Anion
	A:VAL236:CG1 - N:UNK1	3.49805	Hydrophobic	Pi-Sigma
	A:VAL300:CG1 - N:UNK1	3.43682	Hydrophobic	Pi-Sigma
	A:VAL300:CG2 - N:UNK1	3.61349	Hydrophobic	Pi-Sigma
	N:UNK1 - A:VAL236	5.2427	Hydrophobic	Pi-Alkyl
	N:UNK1 - A:ILE296	5.20118	Hydrophobic	Pi-Alkyl
	N:UNK1 - A:ILE296	4.98077	Hydrophobic	Pi-Alkyl
	N:UNK1 - A:VAL300	5.327	Hydrophobic	Pi-Alkyl
	N:UNK1 - A:LYS233	5.21063	Hydrophobic	Pi-Alkyl
	N:UNK1 - A:ILE322	4.94739	Hydrophobic	Pi-Alkyl
22β-hydroxylupeol (Compound 3)	A:VAL236 - N:UNK1	5.2962	Hydrophobic	Alkyl
	A:ILE296 - N:UNK1	4.97496	Hydrophobic	Alkyl
	A:ILE322 - N:UNK1	4.51586	Hydrophobic	Alkyl
	N:UNK1 - A:MET151	4.97485	Hydrophobic	Alkyl
	N:UNK1:C - A:VAL300	3.50406	Hydrophobic	Alkyl
	N:UNK1:C - A:MET151	4.84965	Hydrophobic	Alkyl
	N:UNK1:C - A:ILE296	4.30199	Hydrophobic	Alkyl
	N:UNK1:C - A:ILE144	4.61994	Hydrophobic	Alkyl
	N:UNK1:C - A:CYS217	3.9004	Hydrophobic	Alkyl
	N:UNK1:C - A:VAL143	5.37249	Hydrophobic	Alkyl
	A:HIS54 - N:UNK1	4.83328	Hydrophobic	Pi-Alkyl
	A:TRP133 - N:UNK1:C	4.62235	Hydrophobic	Pi-Alkyl
	A:TYR148 - N:UNK1	4.70752	Hydrophobic	Pi-Alkyl
	A:TRP293 - N:UNK1:C	5.31726	Hydrophobic	Pi-Alkyl
	A:HIS297 - N:UNK1:C	4.31287	Hydrophobic	Pi-Alkyl
β-sitosterol (Compound 4)	A:ASN127:HD22 - N:UNK1:O	2.44148	Hydrogen Bond	Conventional Hydrogen Bond

	A:SER55:CB - N:UNK1:O	3.46491	Hydrogen Bond	Carbon Hydrogen Bond
	N:UNK1:C - A:GLN124:OE1	3.29509	Hydrogen Bond	Carbon Hydrogen Bond
	N:UNK1:C - A:HIS297	3.83839	Hydrophobic	Pi-Sigma
	A:ILE322 - N:UNK1	4.48214	Hydrophobic	Alkyl
	N:UNK1 - A:ILE322	5.25573	Hydrophobic	Alkyl
	N:UNK1:C - A:MET151	5.22846	Hydrophobic	Alkyl
	N:UNK1:C - A:ILE296	3.68992	Hydrophobic	Alkyl
	N:UNK1:C - A:LYS233	4.61256	Hydrophobic	Alkyl
	N:UNK1:C - A:VAL236	4.23074	Hydrophobic	Alkyl
	N:UNK1:C - A:VAL300	4.95085	Hydrophobic	Alkyl
	N:UNK1:C - A:LYS233	4.05313	Hydrophobic	Alkyl
	N:UNK1:C - A:VAL236	3.7841	Hydrophobic	Alkyl
	N:UNK1:C - A:VAL300	4.03548	Hydrophobic	Alkyl
	A:HIS54 - N:UNK1:C	4.37758	Hydrophobic	Pi-Alkyl
	A:TRP293 - N:UNK1:C	4.95598	Hydrophobic	Pi-Alkyl
Morphine	A:TYR148:HH - N:UNK1:O	3.02542	Hydrogen Bond	Conventional Hydrogen Bond
	A:VAL300 - N:UNK1	5.24018	Hydrophobic	Alkyl
	A:HIS54 - N:UNK1	5.38068	Hydrophobic	Pi-Alkyl
	N:UNK1 - A:VAL236	4.94157	Hydrophobic	Pi-Alkyl
	N:UNK1 - A:VAL300	4.30874	Hydrophobic	Pi-Alkyl

Table S8. Molecular docking analysis of the isolated compounds (1 to 4) from *Wrightia coccinea* and the standard compound diclofenac against the cyclooxygenase 2 (COX 2) enzyme (PDB ID: 1XC2).

Compounds	Bond (AA and Ligand)	Bond length (Å)	Bond type	Bond nature
3β-acetyloxy-olean-12-en-28-ol (Compound 1)	A:LYS79 - N:UNK1	4.589	Hydrophobic	Alkyl
	A:LYS79 - N:UNK1	4.99889	Hydrophobic	Alkyl
	A:LYS83 - N:UNK1	5.14197	Hydrophobic	Alkyl
	A:LYS83 - N:UNK1	5.44478	Hydrophobic	Alkyl
	N:UNK1:C - A:ARG44	4.41571	Hydrophobic	Alkyl
	N:UNK1:C - A:LYS83	4.87206	Hydrophobic	Alkyl
	N:UNK1:C - A:PRO84	4.48325	Hydrophobic	Alkyl
	N:UNK1:C - A:LYS83	4.74239	Hydrophobic	Alkyl
	N:UNK1:C - A:PRO84	5.2296	Hydrophobic	Alkyl
	A:PHE64 - N:UNK1:C	5.47654	Hydrophobic	Pi-Alkyl
	A:TYR122 - N:UNK1	4.92815	Hydrophobic	Pi-Alkyl
	A:TYR122 - N:UNK1:C	4.58205	Hydrophobic	Pi-Alkyl
	A:TYR122 - N:UNK1:C	5.31253	Hydrophobic	Pi-Alkyl
Wrightiadione (Compound 2)	A:ASN39:HD21 - N:UNK1:O	3.00244	Hydrogen Bond	Conventional Hydrogen Bond
	A:CYS47:HN - N:UNK1:O	2.13213	Hydrogen Bond	Conventional Hydrogen Bond

	A:GLN461:HE21 - N:UNK1:O	2.26425	Hydrogen Bond	Conventional Hydrogen Bond
	A:GLN461:HE21 - N:UNK1:O	2.78612	Hydrogen Bond	Conventional Hydrogen Bond
	A:GLN461:HE22 - N:UNK1:O	2.61357	Hydrogen Bond	Conventional Hydrogen Bond
	A:MET48:SD - N:UNK1	5.94522	Other	Pi-Sulfur
	N:UNK1 - A:CYS36	5.15783	Hydrophobic	Pi-Alkyl
	N:UNK1 - A:CYS47	5.2364	Hydrophobic	Pi-Alkyl
	N:UNK1 - A:PRO153	4.41136	Hydrophobic	Pi-Alkyl
	N:UNK1 - A:PRO153	4.377	Hydrophobic	Pi-Alkyl
	N:UNK1 - A:CYS36	4.12745	Hydrophobic	Pi-Alkyl
	N:UNK1 - A:CYS47	5.0713	Hydrophobic	Pi-Alkyl
	N:UNK1 - A:PRO153	5.33942	Hydrophobic	Pi-Alkyl
	N:UNK1 - A:PRO153	5.46118	Hydrophobic	Pi-Alkyl
22β-hydroxylupeol (Compound 3)	A:LYS79 - N:UNK1	4.51692	Hydrophobic	Alkyl
	A:LYS83 - N:UNK1	4.72335	Hydrophobic	Alkyl
	N:UNK1:C - A:LEU80	4.5882	Hydrophobic	Alkyl
	A:PHE64 - N:UNK1	5.33052	Hydrophobic	Pi-Alkyl
	A:PHE64 - N:UNK1:C	4.85824	Hydrophobic	Pi-Alkyl
	A:TYR122 - N:UNK1	5.19571	Hydrophobic	Pi-Alkyl
β-sitosterol (Compound 4)	A:ASN34:HD21 - N:UNK1:O	2.26854	Hydrogen Bond	Conventional Hydrogen Bond
	A:CYS36 - N:UNK1	4.58456	Hydrophobic	Alkyl
	A:CYS47 - N:UNK1	4.98618	Hydrophobic	Alkyl
	A:CYS47 - N:UNK1	5.42718	Hydrophobic	Alkyl
	A:PRO153 - N:UNK1	5.42715	Hydrophobic	Alkyl
	A:PRO153 - N:UNK1	4.16156	Hydrophobic	Alkyl
	A:PRO153 - N:UNK1	4.25287	Hydrophobic	Alkyl
	A:PRO153 - N:UNK1	4.90508	Hydrophobic	Alkyl
	N:UNK1:C - A:CYS36	3.97061	Hydrophobic	Alkyl
	N:UNK1:C - A:CYS47	3.62884	Hydrophobic	Alkyl
	N:UNK1:C - A:LEU152	3.96485	Hydrophobic	Alkyl
	N:UNK1:C - A:ARG469	3.87404	Hydrophobic	Alkyl
	N:UNK1:C - A:LYS468	4.0008	Hydrophobic	Alkyl
	N:UNK1:C - A:LYS468	5.35095	Hydrophobic	Alkyl
Diclofenac	N:UNK1:H - A:ASN43:OD1	2.01384	Hydrogen Bond	Conventional Hydrogen Bond
	N:UNK1:H - A:SER471:OG	1.91196	Hydrogen Bond	Conventional Hydrogen Bond
	A:LYS468:NZ - N:UNK1	4.20853	Electrostatic	Pi-Cation
	A:LYS468:C,O;ARG469:N - N:UNK1	3.7266	Hydrophobic	Amide-Pi Stacked
	N:UNK1 - A:ARG44	5.44793	Hydrophobic	Pi-Alkyl
	N:UNK1 - A:LYS468	4.68767	Hydrophobic	Pi-Alkyl

	N:UNK1 - A:LYS468	4.44141	Hydrophobic	Pi-Alkyl
	N:UNK1 - A:PRO474	5.26866	Hydrophobic	Pi-Alkyl
